# Novel Markov model of induced pluripotency predicts gene expression changes in reprogramming

**DOI:** 10.1186/1752-0509-5-S2-S8

**Published:** 2011-12-14

**Authors:** Zhirui Hu, Minping Qian, Michael Q Zhang

**Affiliations:** 1 Department of Molecular and Cell Biology, Center for Systems Biology, the University of Texas at Dallas, 800 West Campbell Road, RL11 Richardson, TX 75080-3021, USA; 2 MOE Key Laboratory of Bioinformatics and Bioinformatics Div, TNLIST /Department of Automation, Tsinghua University, Beijing 100084, China; 3 School of Mathematics, Peking University, Beijing 100871, China

## Abstract

**Background:**

Somatic cells can be reprogrammed to induced-pluripotent stem cells (iPSCs) by introducing few reprogramming factors, which challenges the long held view that cell differentiation is irreversible. However, the mechanism of induced pluripotency is still unknown.

**Methods:**

Inspired by the phenomenological reprogramming model of Artyomov et al (2010), we proposed a novel Markov model, stepwise reprogramming Markov (SRM) model, with simpler gene regulation rules and explored various properties of the model with Monte Carlo simulation. We calculated the reprogramming rate and showed that it would increase in the condition of knockdown of somatic transcription factors or inhibition of DNA methylation globally, consistent with the real reprogramming experiments. Furthermore, we demonstrated the utility of our model by testing it with the real dynamic gene expression data spanning across different intermediate stages in the iPS reprogramming process.

**Results:**

The gene expression data at several stages in reprogramming and the reprogramming rate under several typically experiment conditions coincided with our simulation results. The function of reprogramming factors and gene expression change during reprogramming could be partly explained by our model reasonably well.

**Conclusions:**

This lands further support on our general rules of gene regulation network in iPSC reprogramming. This model may help uncover the basic mechanism of reprogramming and improve the efficiency of converting somatic cells to iPSCs.

## Background

In embryonic stem cells (ESCs), the promoters of *Oct4*, *Sox2* and *Nanog* can be bound by their own products together or separately and an auto feedback loop forms. They also can activate other pluripotent genes and inhibit lineage specific genes. In this way, embryonic stem cell state is reinforced [1]. Differentiated cells are reprogrammed to induced-pluripotent stem cells (iPSC) by ectopic expression of factors which induce the reestablishment of transcription regulation in embryonic stem cell state.

However, up to now, the reprogramming efficiency is still low and the mechanism of reprogramming is not fully understood. In order to enhance the reprogramming rate and reduce the reprogramming latency, the changes of gene expression and epigenetic modifications in the reprogramming process [2,3] and their differences among somatic cells, iPSCs and ESCs [4] are studied extensively, showing that epigenetic modifications and gene expression change dramatically during reprogramming. In addition, epigenetic modification (e.g. DNA methylation and histone modification) plays an important role in development. Knockout experiments show that the deletion of DNA methyltransferase or histone modifiers leads to embryonic lethality. Loss of such epigenetic modifications in ESCs will affect cell differentiation [5]. As the epigenetic landscape shows dynamic change during differentiation and reprogramming, we considered not only the gene expression but also epigenetic modifications in our model to study the basic principles in reprogramming, which may serve as an important medium for gene expression change in reprogramming.

Several models have been established to explain the phenomena in reprogramming, standing to help improve reprogramming efficiency. For example, MacArthur et al. (2008) established a set of differential equations according to the transcription regulatory network in ESC and found that differentiated cells can achieve the iPSC state by amplifying the transcription fluctuation globally [6]. Furusawa et al. proposed that the trajectory in the gene expression phase space is chaotic in the stem cell state, while as the cell differentiates, the complexity of the trajectory decreases. They inferred that the differentiated cells might be reprogrammed by increasing the diversity of expressed proteins [7,8].

Distinguished from these dynamic equation models, Waddington depicted that cell differentiation is like a ball rolling down the hill in the epigenetic energy landscape. The reprogramming process is just the opposite by inducing a set of reprogramming factors (such as Oct3/4, Sox2, c-Myc and Klf4 [9]) to push the system going up with positive probability. Although all the cells have the potency to be reprogrammed, only the cells having overcome all the epigenetic barriers can be reprogrammed to the iPSC state, which depends on some stochastic events with small probability and thus explains the low efficiency of reprogramming. This is the “stochastic model” by Yamanaka (2009), opposite to the “elite model” in which only a small portion of cells can be reprogrammed [10]. Artyomov et al. (2010) developed an Ising model taking account of several general rules governing the interaction between the cell type specific genes [11], which can be used to simulate the rare and stochastic event of successful reprogramming. Most of these rules are crucial and may reveal the underlying principles of cell differentiation and reprogramming; whereas others are redundant and are lack of experimental support. We developed a stepwise reprogramming Markov (SRM) model based on some of the modified rules, which can partly explain gene expression changes, morphology changes and the barriers in the induced pluripotent process.

In this paper, we support the point that cells achieve the pluripotent state gradually through several ordered and well-defined stochastic events [2,3] and some of them happen with small probability, as the epigenetic state in some regions of genome are always hard to be converted to an embryonic stem cell like state [4]. By our model, we also showed that different types of cells have different potential in reprogramming, as less differentiated cells can achieve the iPSC state easier [12].

## Results

### Cell lineage tree, module tree and cell state

**Figure 1:**
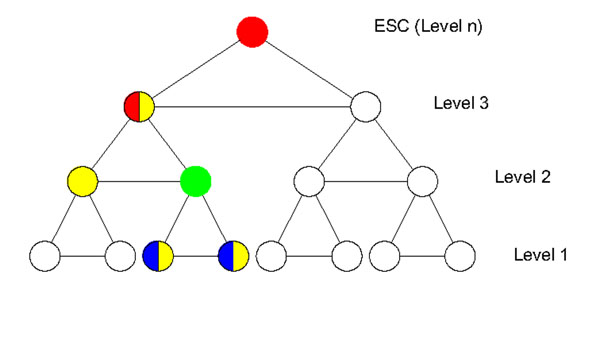
**Module Tree.** Standing at the green module, we see that modules having blue color are descendents of it; modules having yellow color are neighbors of it and modules having red color are progenitors of it.

We assumed that as the cell differentiates, the number of cell types increases exponentially, forming a binary tree. Here we selected groups of cell type specific genes, called modules, for all cell types in the cell lineage tree and arranged them into a module tree with the same hierarchical structure as the cell lineage tree. We denoted neighbors of module A as N_a_ , a set including its sibling, children and parent module; descendents of module A in set P_a_ and progenitors of module A in set Q_a_ (a set of all progenitor modules up to ESC) (Fig 1). We identified the cell type with its corresponding gene module as genes in a module only highly express in the corresponding cell type and have similar behavior in reprogramming. For example, ESC can be represented by a module including *Oct4*, *Nanog*. They highly express in ESC but express low in other cell types (see Additional file 1).

**Figure 2:**
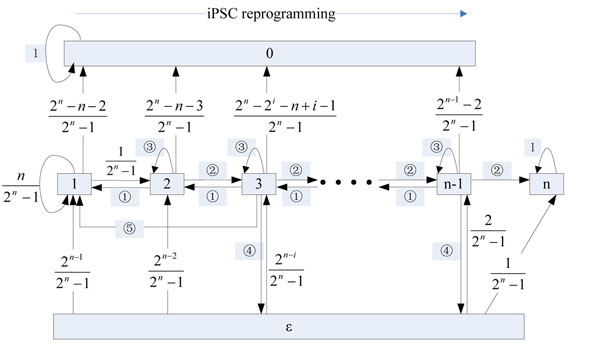
Reprogramming Markov Chain

The cell experiences dramatic change of gene expression and epigenetic patterning during reprogramming. As the expression of the modules sequentially turn on and their epigenetic states temporarily change from “closed” to “open”, we assumed that the cell gradually transits from a more to a less differentiated level in the reprogramming process [3,13]. We defined that a cell is in state ***k*** (or cell in level k) if the cell assumes one of the cell types in the K^th^ level of the cell lineage tree. The embryonic stem cell state is state***n***, while the initial differentiated cell is in state ***1***. When the cell transits from state***1*** to state***n*** after many steps, the cell will stay at state ***n*** in the suitable culture condition since endogenously expressed pluripotency genes can reinforce their expression [1]. However, specific genes of different cell types may express together; in this case, we cannot say which cell type on the cell lineage tree the cell is in. We denoted this state as partially reprogrammed state, ***ε***(detailed definition see below). On the other hand, expression of modules in different lineage may be in conflict with each other, disrupt cell’s transcriptional regulatory network and finally leads to cell death, denoted by state ***0***. Then we considered a Markov chain transit among the states ***0***, ***1***, ***2***… ***n*** and ***ε ***in which state ***0*** and ***n*** are two absorbing states (Fig 2).

Each cell type or state is characterized by a particular combination of the genetic and epigenetic state of all modules. Since the genetic and epigenetic state of all the genes in a module change in the similar manner in reprogramming, we defined the genetic state and epigenetic state of a module by a single value respectively:  with

measures the epigenetic state and genetic state of module i at time k respectively. We defined a cell in cell type  if only . For example, in ESCs, the pluripotency genes in ESC module enrich for H3K4me and are depleted of DNA methylation, inferring the open epigenetic state [5] (Fig S1(a), (b)); while other modules are not open (Fig S1(c)). We defined cell death (state ***0***) as all of the modules in a cell does not express and defined partially reprogrammed state (state ***ε***) if more than one module is in open epigenetic state.

### Rules of modules’ states transition

**Figure 3:**
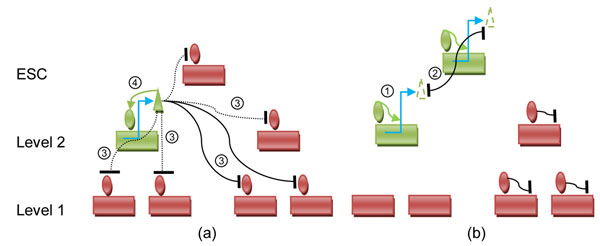
**Transcriptional regulatory Network** (a) RULE1 (b) RULE2. Green rectangle: expressed gene; Red rectangle: repressed gene; Green circle: active chromatin state; Red circle: repressed chromatin state; Triangle: protein. Dash Triangle: little mount of protein. The black curve indicates repression with dash line indicating the effect is not strong. The green arrow indicates activation. RULE1 and RULE2 are represented by ①~④.

In order to determine the transition probabilities between states in the Markov chain above, we first considered the state transition of modules in reprogramming. We focused on two general phenomenological gene regulation rules deduced in the light of Artyomov et al. (2010) where six rules governing transcriptional regulation in cell differentiation and reprogramming were summarized: (a) the epigenetic state of a gene affects its expression (b) gene expression auto-regulates its epigenetic state (c) expression of sibling modules repress each other (d) expression of a module puts inactive epigenetic marks on its progenitor and sibling modules (e) expression of a module puts bivalent epigenetic marks on its progeny (f) expression of a module put negative epigenetic marks on modules on other lineage and upper levels [11]. However, rule (d) and rule (e) may be deduced by the others. Instead, rule (a), (b), (c), (f) are relatively fundamental and supported by a lot of experiments. Based on these four rules, we established our SRM model with two simplified rules:

RULE1: effect on epigenetic state by gene expression. Expression of a module makes its epigenetic state open (for example, the auto-activation loop of *Oct4*, *Nanog* and *Sox2* mentioned above) [1] (shown as ④ in Fig 3). It also makes its neighbors or non-descendent modules close (shown as ③ in Fig 3), which maintains the identity of the cell and prevents cell differentiation. Besides, the repression dominates the auto-activation when they coexist [14]. Since the repression strength gets weaker as the distance in the module tree gets shorter, the repression by neighbor modules will be cancelled out by auto-activation. One can compute the state of module at the next time point, k+1, as follows:(1)

RULE2: effect on gene expression by epigenetic state. When the epigenetic state of a module is open, the module express otherwise the module doesn’t express (shown as ① in Fig 3). If more than one module is in open epigenetic state, they will express low as they repress each other and the cell gets to the partially reprogrammed state (shown as ② in Fig 3). It also shows that sometimes some genes in different lineages express together in reprogramming [3], which can be represented by state ***ε***. Thus, one could compute the state of module at the next time point, k+1, as follows:(2)

Noticing that in the update by (1) for epigenetic states all genetic states never change, one can see that the update process does not depend on the order of selection for *i.* After doing (1) for all *i*, do (2) for all *i*, called a round, in phase with one cell cycle, and repeat until the cell gets to the *equilibrium state* when all the modules are invariant under RULE1 and RULE2. In *equilibrium state*, the cell will be in one of the cell types, death or partially reprogrammed state (see Additional file 1).

### The role of the reprogramming factors and procedure in SRM model

The reprogramming factors can bind to genes associated with differentiation or pluripotency, repressing or activating gene transcription respectively. In ESC, the repression effect of the reprogramming factors is related with the recruitment of repressive chromatin remodeling complexes such as NuRD and Polycomb, resulting in histone deacetylation and H3K27 trimethylation [12]. On the other hand, downregulation of somatic markers is related with c-Myc mostly, which has significant effect in the early reprogramming and seems more likely to bind genes with accessible chromatin state [15]. Taking into account these phenomena, we simulated the reprogramming factors repression as choosing a module with open or bivalent epigenetic state (not 0) randomly and making it close.

To simulate the activation of the reprogramming factors, as most of genes chosen in modules are regulated by the reprogramming factors, a module is chosen randomly with equal probability regardless of which epigenetic state it is in (many reprogramming factor binding targets in ESCs, iPSCs or partially reprogrammed cells have repressive histone modification markers [15]), made express and made its chromatin state open. However, in reality, the reprogramming factors are more likely to activate some specific genes, which is similar with knockdown of some specific transcription factors and thus the reprogramming factors can’t induce them. The consequence is shown below.

Further, transcriptional repression is always associated with a single reprogramming factor binding. In contrast, when bound by multiple reprogramming factors, gene will be actively transcribed since basal transcriptional machinery is recruited [12]. When the module chosen to be repressed happens to be the same as the one to be activated, the module will express and the epigenetic state of the module will be open.

In sum, if the reprogramming factors repress module A, then: since some protein still remains in the cell. If the reprogramming factors activate module A, then: . If reprogramming factors activate and repress module A at the same time, then: . In particular, if the cell is in state***ε***, the reprogramming factors will activate a module randomly and the cell leaves state***ε***. The cell can get out of the partially reprogrammed state when cultured for longer time [3].

In the reprogramming process, the reprogramming factors will change the epigenetic state and/or expression of the module. Then, in one round, the epigenetic state of each module will be further changed by the protein content in the cell according to RULE1 and the new epigenetic state will change the module expression following RULE2. After two rounds, the cell gets to the equilibrium state (see Method). Then, the reprogramming factors take its effect again unless the cell is in the absorbing state. Thus, the induction of the reprogramming factors will take place every other round. The procedure is shown below:

i. Initially, assume the cell is in type  in the K^th^ level.

ii. The effect of the reprogramming factors is to activate a module and repress one.

iii. In one round:

∀i ∈ {1, 2⋯2^n^ – 1}, S^i^ is changed according to the rule (1).

∀i ∈ {1, 2⋯2^n^ – 1}, G^i^ is changed according to the rule (2).

iv. Repeat iii once. Then, the cell would reach an equilibrium state. Record the cell state transition.

v. Stop if cell reaches iPS state or dies, otherwise go to ii.

Then, we could estimate all the transition probabilities every two rounds in the Markov chain (see method and Fig 2).

### Estimating reprogramming rate and average reprogramming time

We defined a vector , x_i_ is the probability the cell in state ***i, ***i=1…n, 0, ε. Define P as the transition matrix of the Markov chain. Suppose that all of the cells are in state ***i*** initially, . As the number of rounds 2k goes to infinity, , in which p_n_ is the proportion of cells successfully reprogrammed and d_n_ is the death rate. Suppose there are 4 levels in the cell lineage tree. Then the reprogramming rate is 0.02% when reprogrammed from the first level (Table 1), which is consistent with the real experiment where the reprogramming rate is about 0.001%~1% [12]. We estimated the reprogramming rates in the case of other number of levels, which are roughly in the range above when there are not too many levels between the initial cell type and ESC (see Table S1). Our simulation also showed that the reprogramming rate for cells from different differentiated level is different. Further, we calculated the average time needed for reprogramming by computing the expectation of time arriving in state ***n*** (n=4) on the condition that the cell is still alive: Expectation (cell cycles needed for reprogramming |can be reprogrammed) = 8.72 cell cycles.

**Table 1:** Reprogramming rate and average reprogramming time of cells from different levels

Initial cell type	First level	Second level	Third level	Fourth level
**Reprogramming rate (SRM model)**	0.024%	0.26%	3.13%	100%
**Death rate**	99.98%	99.74%	96.87%	0%
**Average reprogramming time (cell cycles)**	8.72	6.00	3.42	0

When knockdown of somatic transcription factors, reprogramming rate from first level is 0.027% and average reprogramming time is 7.16 cell cycles. In reprogramming Ising model, reprogramming rate from first level is 0.025%.

**Figure 4:**
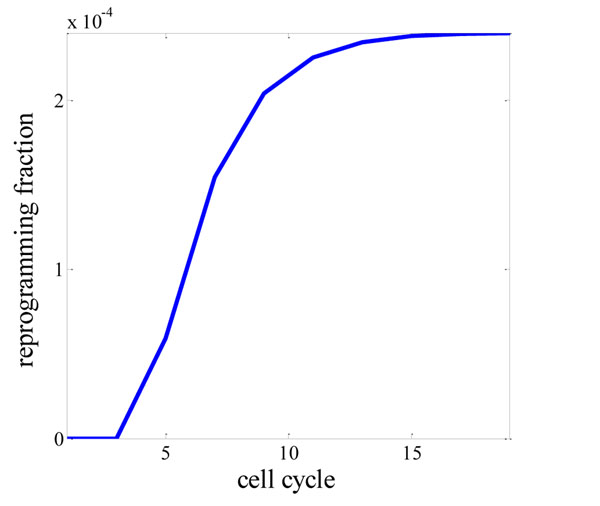
Number of Cells successfully reprogrammed increases in the sigmoid Pattern

From p_n_(k), the portion of cells reprogrammed after 2k rounds (cell cycles), it shows that the number of successfully reprogrammed cells increase in the sigmoid pattern (Fig 4). It takes 6 cell cycles to reach the half of the maximum (response time). As the reprogramming is achieved for some time, the epigenetic states of more modules are not closed, since the module in the upper level is already open. Thus, the repression effect of the reprogramming factors is diminishing, as also observed experimentally that withdrawal of c-Myc after day 5 would not affect reprogramming rate [15].

### Simulating reprogramming in the condition of somatic transcription factors knockdown or DNA methylation inhibition

In the condition of knocking-down somatic transcription factors, the reprogramming rate would be a little higher, about 0.03%. The cell can get to the iPS state faster with the average time 7.16 cell cycles and the response time 5 cell cycles (see Additional file 1 and Fig S2(a)). Also, it has been shown that knock down of *Pax5* will improve the reprogramming efficiency of B cell [12]. Moreover, when treating the cells with DNA methyltransferase inhibitor which attenuates the global repression of DNA methylation, reprogramming efficiency accelerates a lot, about 1.1% in 20 cell cycles (see Additional file 1 and Fig S2(b)). Previous experiments demonstrate that inhibition of Dnmt1 can improve reprogramming efficiency [3].

### Simulating gene expression changes in reprogramming

Suppose all of the cells are in state ***1*** initially, namely . We calculated the expression of modules in different level in terms of cell cycles (or approximately reprogramming days). We defined the total expression of all modules in K^th^ level at time t as the portion of living cells arrived at state ***k*** at time t:(3)

**Figure 5:**
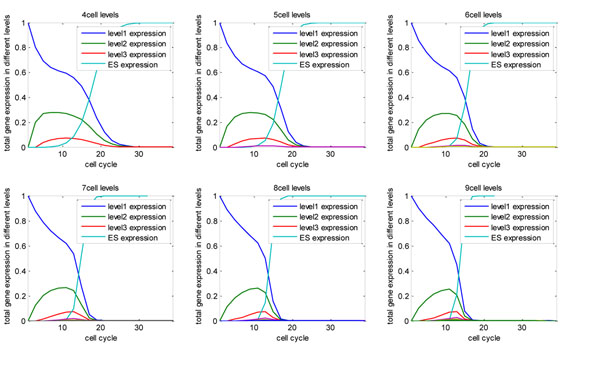
**Change of gene expression is independent of how many Levels in the module tree.** Assuming n=4~9, the expression of genes in level1, level2, level3 and ESC is depicted in blue, green, red, light blue respectively, while the expression of genes in other levels are nearly zero all the time.

The gene expression of modules in level 1 decreases dramatically at the beginning of the reprogramming, as the reprogramming factors inhibit the expression of somatic genes. Next, some genes from upper level express transiently but the cell can’t stay at the de-differentiated state, so it drops back to state ***1***, leading to a period when the gene expression in level 1 does not change much. Finally, more and more living cells get to the ESC state and stay there stably, so expression of somatic genes drops rapidly again and decreases to zero. The expression of ESC’s specific genes increases at last, after the dramatic drop of somatic cell gene expression and the peak time of genes from other levels, which agrees with the real experiment that the endogenously expression of ESC specific genes is the last step in reprogramming. In the early time point, even if pluripotency genes are activated by the reprogramming factors, their chromatin state may close again when inhibited by other modules. The auto-regulation loop cannot form, so pluripotency genes express transiently and their expression doesn’t show any increase in our equilibrium state curve. Moreover, the reprogramming factors only bind transiently and show weak binding strength in intermediate reprogramming cells than ESCs or iPSCs, as observed in [15]. Only in the late period when repression by other modules attenuates can the ESC specific genes continuously express, which may explain the expression curve of ESC specific genes and the long latency of reprogramming. The expression of genes in all other levels first rise then drop although their peak times are different. The genes in level 2 get to peak earlier than level 3 (at cell cycle 7 and 12, respectively). Only genes in these two levels express in a large amount in reprogramming although total number of cell levels are different. As cell levels increases, gene expression changes more dramatically as all the curves become steeper (Fig 5). The tendency of gene expression change in our model mimics the real data roughly (Fig 7(b)).

**Figure 6:**
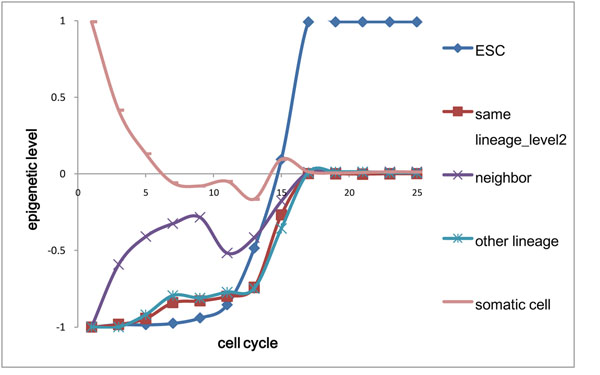
**The epigenetic State of each Node in each Cell Cycle**. Same lineage in level 2, other lineage and neighbor are relative to the initial somatic cell.

**Figure 7:**
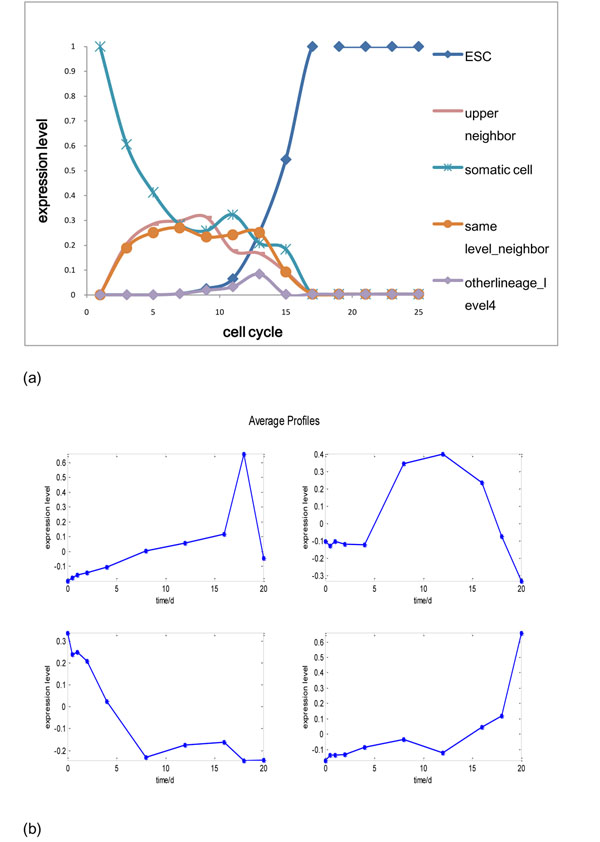
**The average Profiles of real Data match Gene Expression simulated by the reprogramming Ising Model** (a) The genetic state of each node at each cell cycle in reprogramming Ising model. Neighbor in the same level, other lineage in level 4 and upper neighbor are relative to the initial somatic cell. (b) The standardized average profiles of gene expression in each cluster at each time point or in partially reprogrammed cells. Genes in each node can be assigned to a cluster by similar gene expression pattern. Upper left, first cluster. Upper right, second cluster. Lower left, third cluster. Lower right, fourth cluster.

Although some stochastic events will affect cell reprogramming, reprogramming follows a series of defined steps [12]. In our model, the somatic cell de-differentiates level by level and gene expression change follows the same steps as observed in experiment. After ESC specific genes express, still some adjustments of epigenetic state of other modules are needed to get to the equilibrium state, leading to a state more similar to ESC state, as observed that there are some differences in DNA methylation in early cultured and late cultured iPSCs [12].

From the simulated gene expression, our model can track cell type transition in reprogramming. For example, in reprogramming of MEF, fibroblast will change to tightly arranged round cells, inferring that mesenchymal to epithelial transition (MET) takes place [13]. The reprogramming model can simulate MET in reprogramming process (the results are omitted due to space limitation, see Additional file 1). These results may verify the existence of MET and a series of cell type transformations toward less differentiated cell types during reprogramming.

### Reprogramming Ising Model

In order to simulate the fluctuations in reprogramming, we computed the cell “energy” according to the epigenetic and genetic interactions between different modules based on a related model similar to Artyomov’s model in [11], so that module state is changed approximately by (1) and (2) when the perturbation is small. Construct the four level cell lineage binary tree, but ESC in the first level and initial somatic cell in the fourth level. The state of each node in the corresponding module tree contains the genetic and epigenetic state of specific genes of such cell type. G=0,1 representing the expressed and silenced genetic state respectively; while S=-1, 0, 1 representing the closed, bivalent and open epigenetic state respectively. Then the cell “energy” is defined based on the interaction between genetic state and epigenetic state of the nodes (see Method). At every other cell cycle, the reprogramming factors, following the same rule as in SRM model, will activate a node to make its epigenetic state open [11] while repress a node with open epigenetic state to make its epigenetic state close randomly. Then the cell reaches equilibrium state which can be found by Monte Carlo simulations and cell type may change. There are much more varieties of cell states than that in the SRM model. However, the SRM model captures major cell states in reprogramming [12].

We simulated 20000 cells for 25 cell cycles with 5 cells successfully reprogrammed. The reprogramming rate is 0.025%, consistent with rare reprogramming event. The successfully reprogrammed cells reach the ESC state in 8~12 cell cycles (Fig S9), much shorter than the time we can detect them, since it takes some time for the total expression of pluripotent genes increasing to reach the detection threshold as successfully reprogrammed cells divide faster and others die. We averaged the epigenetic and genetic state of each node in each cell cycle of the 20000 cells (shown as Fig 6, 7(a)). The epigenetic state of initial cell type node changes from open to closed and arrives at bivalent state finally (an example in Fig S10). The epigenetic state of ESC node changes from closed to open, during which change occurs rapidly from the 11^th^ cell cycle and on (some examples in Fig S11). From 13^th^ cell cycle and on, the epigenetic state of other nodes begins to change rapidly from closed to bivalent state. In ESC, many genes related to differentiation are in bivalent state [16]. The time point of these three situations agrees with the observation that initial epigenetic change confines within genes with open epigenetic mark in somatic cell and the repression markers are lost later on [2]. We also found that the cell “energy” increases during the reprogramming process, because of less repressive histone markers and thus repression potential H_3_ (see Method) is less. In vivo, it is also known that pluripotent cells have the lowest DNA methylation level [5].

The expression of initial cell type node decreases rapidly in the first several cell cycles; the expression of ESC node increases rapidly from 11^th^ cell cycle on; the expression of upper level neighbor and sibling node first increases and then decreases; the expression of nodes in lineages other than that of the initial cell type remain near zero. However, some nodes in other lineages may be weakly expressed during late reprogramming and may represent the partially reprogrammed state.

Then, we did Kmeans clustering of the real gene expression data in reprogramming (see method). The gene can be divided into 4 groups. The average profile of each cluster matches the gene expression curve predicted in the reprogramming Ising model (Fig 7(b)). The expression change of the first cluster resembles the nodes in other lineages. We found that the cluster is enriched in RNA processing and RNA splicing (Fold enrichment>2, Benjamini<10^-4^). Besides, the genes related with other lineage (e.g. nuclear protein *Ldb1a* related to hemopoietic stem cell maintenance and erythrocyte formation, endoderm transcription factor *Gata6*, glomerular protein *Podxl*, epidermal protein *Sprr1a*, transcription factor *Pax7* related with neuron and skeleton development and transcription factor *Phox2b* related with neuron development) are in this group.

The expression change in the second cluster resembles nodes of father and sibling. This cluster is enriched in actin filament-based process and actin cytoskeleton organization (Fold enrichment>2, Benjamini<0.05). Besides, the genes that are related to basolateral plasma membrane, adherens junction, mesoderm development (such as *hand2* which can be regarded as in the father node) and embryonic skeletal system development (such as *Flnb* which can be regarded as in the sibling node) are in this group.

The average profile of the third cluster is very similar to the curve of somatic cell node. Both of them arrive at 50% of the initial value at 4^th^ cell cycle. We also did the simulation without considering the repression of reprogramming factor. In that case, the expression of somatic cell node decreases so slowly that arrives at 50% at 9^th^ cell cycle. It may mean that the reprogramming factor has a significant role in repressing somatic specific genes in the early reprogramming. This cluster is enriched in appendage development, skeletal system development and extracellular matrix, such as *biglycan* (Fold enrichment>2, Benjamini<10^-4^), which are specific genes of fibroblast. Besides, fibroblast marker *Thy1* and fibroblast structure genes *Col1a1*, *Col1a2* are also in this group.

The average profile of the last cluster resembles the curve of ESC node. This cluster is extremely enriched in condensed chromosome, chromosome in centromeric region and cell division (Fold enrichment>3, Benjamini<10^-26^). This means that chromatin remodeling may be the major event in late reprogramming and cell obtains the ability of self-renewal gradually. Besides, genes maintaining ESC state or related to self renewal (such as *Oct4*, *Klf5*, *Socs3*, *Sox2*, *Nanog*, *Fgf4*) are in this group.

In summary, the gene expression change predicted by our reprogramming Ising model is consistent with the real time-serial gene expression data in reprogramming.

## Conclusions

In this paper, we attempted to explain observed phenomena in reprogramming by a mathematical model. Based on the model in [11], we simplified the regulation of epigenetic and genetic network into two rules and defined the role of reprogramming factors as repressing and activating specific modules according to papers studying the binding sites and function of these factors [12,15]. The module state and cell state transition are shown more clearly in SRM model than the reprogramming Ising model and thus we could see that the epigenetic barriers are created by the expression of modules in lower levels and other lineage. Therefore, we may design more efficient way to overcome these barriers. We simulated the trajectory of rare reprogrammed cell and showed that knockdown of somatic transcription factors or inhibition of DNA methylation or other repressive histone markers can accelerate reprogramming. The SRM model can predict the gene expression change in MET and Ising model can predict the expression change of cell type specific genes, which provides support for these models and the proposed underlying rules governing epigenetic and genetic regulatory network in the cell. Reprogramming is a battle between the reprogramming factors and the cell’s intrinsic transcriptional network. Cells can only reprogram gradually in several ordered and defined steps because of the inhibition of intrinsic interaction. The probability that the cell can overcome all the barriers is very small. Thus, the reprogramming efficiency is often very low. Besides adding specific transcription factors of desired cell type, the intrinsic network in the original cell must be disrupted either by global fluctuation or knocking down specific transcription factors in order to convert itself to the desired cell type.

The two models construct a blue picture of cell type conversion and may be used to study the cell type conversion between different differentiated lineage and transformation between cancer and normal cells. They may be used to identify significant factors in cancer development, for example EMT mentioned in the paper is related to metastasis of cancer cells.

However, these two models are simple. They can only simulate the gene expression and epigenetic change qualitatively but not quantitatively. We neglected some degree of heterogeneity between modules. For example, the expression level of different modules in “on” state is not the same; the probability distribution of the reprogramming factors inducing different modules may be non-uniform. Although these models can provide some insight into the effect of adding histone modifiers or DNA demethylase, knocking out apoptosis factors or somatic cell specific transcription factors, only by taking account of particular gene regulation network can these models help experiment design. Meanwhile, the rules in the model need further experimental verification. As the development of single cell RNA-seq [17] and ChIP-seq advance, the variations between cells can be revealed. Using new technologies, we can understand the mechanism underlying reprogramming more clearly.

We believe that reprogramming efficiency and the safety of iPSC can be further improved by combing experimental result with modeling. As the mechanism of induced pluripotency is understood more comprehensively, iPSC can be widely used in modern molecular medicine.

## Methods

### Markov model development

Upon the reprogramming factors induction, cell will get to another equilibrium state. We enumerated all possible cell state transitions as the reprogramming factors can induce different modules. We calculated the transition probability between different levels in cell lineage tree, that is, different states in the Markov chain, by counting possible reprogramming factor induction sites leading to such transition and dividing by the total number of modules, assuming the reprogramming factors activate each module with equal probability (for detailed calculation of transition probability, see Additional file 1).

### Reprogramming Ising model development

Cell “energy” contains 4 terms. According to RULE2, H_1_=-D•G_i_•S_i_^cell^ i=1,2…15 (the node is numbered as in Fig S9) depicts the effect of epigenetic state on genetic state and H_2_=E•G_i_•G_j_, (i, j are neighboring nodes), depicts the mutual repression. According to RULE1, H_3_=M• G_i_^cell^ •S_j_, (j is not the descendant of i), depicts gene expression putting negative histone marks on nodes in other lineages or upper levels and H_4_=-N• G_i_^cell^ •S_i_ depicts the effect of genetic state on epigenetic state. Thus, the “energy” of the cell is H=H_1_+H_2_+H_3_+H_4_, G_i_ and S_i_ are the genetic and epigenetic state of node i, respectively, G_i_^cell^ and S_i_^cell^ are the average genetic and epigenetic state of node i in the last step, respectively. In the simulation, we assumed KT=1, E=50, D=20, M=8, N=5, F_1_=4, F_2_=16, so that the cell type transition probability in the reprogramming Ising model is the same as that in the SRM model. The reprogramming factors are like an external field, H_5_=δ(i,a)•F_1_•S_i_-δ(i,b)•F_2_•S_i_, with a and b representing the repressed and activated nodes, respectively.

After the induction of the reprogramming factors, using Metropolis algorithm, S_i_ or G_i_ is adjusted according to transition probability . In one cell cycle, S_i_ is first adjusted by ; then, G_i_ is changed by . After two cell cycles, the reprogramming factors act again. “Temperature” KT sets the average “energy” scale and represents the base-level transcription rates. If the sum of all the G_i_ is smaller than 0.01, which is about the fluctuation of G_i_, cell dies and exits the simulation. If the genetic state of ESC node is 1 while others are less than 0.01, the reprogramming factors will be withdrawn. As observed in the experiment, cell cannot be reprogrammed unless the reprogramming factors are withdrawn at the suitable time [12] (for the choice of parameters and the relation of these two models see Additional file 1).

### Expression data

We collected microarray data from GSE10874 (Mikkelsen et al. [3]), GSE26100 (Koche et al. [2]) including gene expression profiles at 0,1,2,4,8,12,16 days of MEF reprogramming and cell lines MCV6, MCV8.1. MCV6 are the partially reprogrammed cells and MCV8.1 is a clone from iPSCs [3]. We used Dchip [18] to normalize the data and did model-based correction (processed data is shown in Additional file 2). Before doing Kmeans clustering, we filtered out 25274 probe sets with Present calls in more than 20% chips and expression level more than 20 in more than 50% chips and standardized the expression data for each gene. Adding the MCV6 expression data between 16 days and MCV8.1, we used correlation as the distance measure in Kmeans clustering. Then we picked the probe sets within 0.1 from the center of each cluster to do gene function enrichment analyses for the cluster using Bioinformative Resource 6.7 [19,20]. There are about 1500~2000 Entrez genes in each of the filtered cluster.

## Competing interests

The authors declare no competing interests.

## Authors' contributions

ZRH designed the model**,** did the simulation and wrote the manuscript. MQZ proposed the project**,** conceived of the paper and revised the manuscript. MPQ revised the manuscript and helped analyzing the model. All authors read and approved the manuscript.

## Supplementary Material

Additional file 1 **Details and application of the model** Including 11 figures and 1 table; Simulating MET in reprogramming; Mathematics details Click here for file

Additional file 2 **Gene expression data** This file includes normalized gene expression data in different days in reprogramming. Click here for file
