# Supplementary material for: Novel Markov model of induced pluripotency predicts gene expression changes in reprogramming
Source: BMC Syst Biol. 2011 Dec 14;5(Suppl 2):S8. doi: 10.1186/1752-0509-5-S2-S8 (PMC3287488; doi:10.1186/1752-0509-5-S2-S8)
Supplement: Additional file 1 — Details and application of the model Including 11 figures and 1 table; Simulating MET in reprogramming; Mathematics details [file 1752-0509-5-S2-S8-S1.docx]

Details and application of the model

## Equilibrium states of a cell

Although there are lots of equilibrium states of a cell under the definition, only two kinds of them occur in the model after operations of (1) and (2):

If the cell assumes cell type A, only module A=$\left( \begin{aligned} 1 \\ 1 \end{aligned} \right)$. Other module, B, since the expression of module A has no effect on its descendents other than children, then:

$B=\left\{ \begin{aligned} \left( \begin{aligned} 0 \\ 0 \end{aligned} \right) B is not the descendent A or is the neighbor of A \\ \left( \begin{aligned} \varepsilon\\ 0 \end{aligned} \right) 0<\varepsilon\ll1 B is in the bivalent state in other cases \end{aligned} \right.$ (S1)

For example, genes in ESC module (*Nanog*, Fig S1 (a)) show high level of H3K4me3 (=$\left( \begin{aligned} 1 \\ 1 \end{aligned} \right)$) in ESCs, but some H3K27me3 (significant peaks, p<10^-10^)$(=\left( \begin{aligned} 0 \\ 0 \end{aligned} \right)$) in other cell types. While genes in somatic cell module (*BGN*, Fig S1 (c)) has some H3K4me3 and H3K27me3 in ESCs and other cell types$(=\left( \begin{aligned} \varepsilon\\ 0 \end{aligned} \right)$); are enriched for H3K4me3 (=$\left( \begin{aligned} 1 \\ 1 \end{aligned} \right)$) in HUVEC (umbilical vein endothelial cells from mesoderm).

If the cell is in partially reprogrammed state, then some modules are $\left( \begin{aligned} 1 \\ \varepsilon\end{aligned} \right)$, others are $\left( \begin{aligned} \varepsilon\\ 0 \end{aligned} \right)$.

## The transition probability in the Markov model

Suppose the cell is in cell type A and in state ***i*** initially, module A is in state $\left( \begin{aligned} 1 \\ 1 \end{aligned} \right)$. **Situation 1**: A is repressed by reprogramming factors with probability$p_{i}=\left\{ \begin{aligned} \frac{1}{2^{i}-3} 1<i<n \\ 1 i=1 \end{aligned} \right.$, since module A and its descendents but not neighbor modules have open chromatin structure and they can be repressed with equal probability. Then for A: $\left( \begin{aligned} 1 \\ 1 \end{aligned} \right)\underset{\to}{\mathrm{RE}}\left( \begin{aligned} 0 \\ 1 \end{aligned} \right)$ and reprogramming factors activate module $B: \left( \begin{aligned} a \\ b \end{aligned} \right)\underset{\to}{\mathrm{AC}}\left( \begin{aligned} 1 \\ 1 \end{aligned} \right)$. The states of A and B change as the major effect of reprogramming factors followed by changes of other modules in order to get equilibrium with the new state of A and B according to (S1). There are 5 different conditions in this situation:

1. $B\in{{(P}_{a}\cup N_{a}\cup A\cup Q_{a})}^{c}$ is in other lineage. As protein expressed from module A or B makes the other’s epigenetic state close by RULE1, $\left( A B \right)=\left( \begin{matrix} 0 & 0 \\ 1 & 1 \end{matrix} \right)$; epigenetic states of other modules change to 0 or ɛ by (1). Then by (2), $\left( A B \right)=\left( \begin{matrix} 0 & 0 \\ 0 & 0 \end{matrix} \right)$. Modules’ genetic states are all zero thus cell will die with probability $P_{i0}^{1}=\frac{2^{n}-2^{i}-n+i-1}{2^{n}-1}\cdot p_{i} i<n$, since there are $2^{n}-2^{i}-n+i-1$ modules in ${{(P}_{a}\cup N_{a}\cup A\cup Q_{a})}^{c}$ and $2^{n}-1$ modules in total.
2. B is neighbor module of A. In the first cell cycle, by (1), $\left( A B \right)=\left( \begin{matrix} 0 & 1 \\ 1 & 1 \end{matrix} \right)$; epigenetic states of other modules change to 0 or ɛ. Then by (2), $\left( A B \right)=\left( \begin{matrix} 0 & 1 \\ 0 & 1 \end{matrix} \right)$. In the next cell cycle, epigenetic states of other modules change to 0 or ɛ as in (S1) and cell can convert to cell type B. In this case, the cell may dedifferentiate one level or stay at the same level or differentiate one level depending on the position of B. If neighbor module in level $i-1$ is activated, $P_{i,i-1}=\frac{2}{2^{n}-1}\times p_{i}1<i<n$. If neighbor module in level $i+1$ is activated, then $P_{i,i+1}=\frac{1}{2^{n}-1}\times p_{i} i<n$.Some experiment shows that by over-expression of transcription factors, cell type conversion happens in a much higher rate than reprogramming, for example, all B cells can switch to macrophages induced by C/EBPα in 48 hours [12]. It means that conversion between somatic cells may not need de-differentiation and then differentiation; instead, the cell may be converted directly. This is just the case we described here. Transcription factors activate the specific genes of desired cell type while repress the module of initial cell type. The cell will convert successfully in two cell cycles. However, the efficiency of cell type conversion between different lineages may be lower for cell has to de-differentiate.
3. $B\in Q_{a}\backslash N_{a}$ in the same lineage as A. In the first cell cycle, by (1), $\left( A B \right)=\left( \begin{matrix} 1 & 0 \\ 1 & 1 \end{matrix} \right)$; epigenetic states of other modules change to 0 or ɛ. Then by (2), $\left( A B \right)=\left( \begin{matrix} 1 & 0 \\ 1 & 0 \end{matrix} \right)$. In the next cell cycle, epigenetic states of other modules change to 0 or ɛ as in (S1) and cell type remains the same. Thus, $P_{\mathrm{ij}}^{1}=0，i+1<j\leq n$.
4. $B\in P_{a}\backslash N_{a}$. In the first cell cycle, by (1), $\left( A B \right)=\left( \begin{matrix} 0 & 1 \\ 1 & 1 \end{matrix} \right)$; epigenetic states of other modules change to 0 or ɛ. Then by (2), $\left( A B \right)=\left( \begin{matrix} 0 & 1 \\ 0 & 1 \end{matrix} \right)$. In the next cell cycle, epigenetic states of other modules change to 0 or ɛ as in (S1) and cell will convert to cell type B. In this case, the cell returns to the level where B is located in. Thus, $P_{\mathrm{ij}}^{1}=\frac{2^{i-j}}{2^{n}-1}\cdot p_{i}，0<j<i-1,2<i<n$.
5. When reprogramming factors activate module A, $\left( \begin{aligned} 0 \\ 1 \end{aligned} \right)\underset{\to}{\mathrm{AR}}\left( \begin{aligned} 1 \\ 1 \end{aligned} \right)$, cell remains in cell type A.

**Situation 2**: A is not repressed by reprogramming factors with probability ${1-p}_{i}$, then $A=\left( \begin{aligned} 1 \\ 1 \end{aligned} \right)$. Again, there are 5 conditions. Since the consequence of module B being both repressed and activated is the same as being activated, the cell type transition is the same as that in **situation 1** except in condition 2. Thus,$P_{i0}^{2}=\frac{2^{n}-2^{i}-n+i-1}{2^{n}-1}\cdot\left( {1-p}_{i} \right) i<n P_{\mathrm{ij}}^{2}=0，i+1<j\leq n$ $P_{\mathrm{ij}}^{2}=\frac{2^{i-j}}{2^{n}-1}\cdot{(1-p}_{i})，0<j<i-1,2<i<n$

For condition 2, when reprogramming factors activate neighbor module B, then for B, $\left( \begin{aligned} a \\ b \end{aligned} \right)\underset{\to}{\mathrm{AC}}\left( \begin{aligned} 1 \\ 1 \end{aligned} \right)$. In the first cell cycle, by (1), $\left( A B \right)=\left( \begin{matrix} 1 & 1 \\ 1 & 1 \end{matrix} \right)$; epigenetic states of other modules change to 0 or ɛ. Then by (2), $\left( A B \right)=\left( \begin{matrix} 1 & 1 \\ \varepsilon& \varepsilon\end{matrix} \right)$. In the next cell cycle, epigenetic states of other modules change ɛ as in (S1) and cell will be in state ***ɛ*** with probability $P_{i,\varepsilon}=\frac{4}{2^{n}-1}\times\left( 1-p_{i} \right) 2<i<n$. The state can only occur when module in the upper levels of the lineage tree turns on, coincident with the fact that partially reprogrammed state is observed in the late stage of reprogramming. Taking together these two situations:

$$P_{i0}=P_{i0}^{1}+P_{i0}^{2}=\frac{2^{n}-2^{i}-n+i-1}{2^{n}-1}, i<n$$

$$P_{\mathrm{ij}}=0，i+1<j\leq n$$

$$P_{\mathrm{ij}}=P_{\mathrm{ij}}^{1}{+P}_{\mathrm{ij}}^{2}=\frac{2^{i-j}}{2^{n}-1}，0<j<i-1,2<i<n$$

$$P_{i,\varepsilon}=\frac{4}{2^{n}-1}\cdot\left( 1-p_{i} \right)=\frac{4}{2^{n}-1}\cdot\left( 1-\frac{1}{2^{i}-3} \right), 2<i<n$$

$$P_{i,i-1}=\frac{2}{2^{n}-1}\cdot p_{i}=\frac{2}{2^{n}-1}\cdot\frac{1}{2^{i}-3}, 1<i<n$$

$$P_{i,i+1}=\frac{1}{2^{n}-1}\cdot p_{i}=\left\{ \begin{aligned} \frac{1}{2^{n}-1}\cdot\frac{1}{2^{i}-3} 1<i<n \\ \frac{1}{2^{n}-1} i=1 \end{aligned} \right.$$

In other circumstances, the cell will remain in state ***i***, then: $P_{i,i}=\left\{ \begin{aligned} {(1+n-i-1+\frac{1}{2^{i}-3})}/{{(2}^{n}-1) 1<i<n} \\ \frac{n}{2^{n}-1} i=1 \end{aligned} \right.$.

The cell will stay in state ***n*** or state ***0*** once it arrives there. $P_{\mathrm{nn}}=1，P_{00}=1$. Suppose the cell is in state ***ɛ***. The reprogramming factor will activate a module randomly. Thus, $P_{ɛ,i}=\frac{2^{n-i}}{2^{n}-1}$. Then we got the transition probability shown in Fig 2 (in the main text).

## Simulating the effect of knockdown of somatic transcription factors and inhibition of DNA methylation

If transcription factors of the initial somatic cell are knocked down, we deleted the module in the simulation after the first two rounds. Suppose the cell is in cell type A initially and module A is in state $\left( \begin{aligned} 1 \\ 1 \end{aligned} \right)$. The state of A changes to $\left( \begin{aligned} 0 \\ 1 \end{aligned} \right)$ after knockdown, which is similar to repression by some factors. In the first two rounds, the scenarios are the same as **situation 1** in the above section except that condition 3 and 5 lead to cell death as the expression of module A is repressed and thus all modules are off. After two cell cycles, module state transition follows the same rules as in the above section except that the reprogramming factors can’t induce module A and cell can’t get to cell type A. By calculating all the transition probability between cell equilibrium states, we got the number of cells successfully reprogrammed in respect of time (Fig S2).

The reprogramming rate improves a little since knockdown of somatic transcription factors promotes dedifferentiation and brings down the probability of cell returning to the first level but cell death increases in the first two rounds as indicted in the model.

On the other hand, when treating the cell with DNA methylation inhibitor, the repression described in RULE1 becomes weaker and is offset by auto-activation. The operation changes to:

First, $S_{k+1}^{i}=\left\{ \begin{aligned} 1 if G_{k}^{i}=1 \\ S_{k}^{i}\mathrm{if}G_{k}^{i}=\varepsilon\\ \varepsilon\mathrm{others} \end{aligned} \right.$; then, $S_{k+1}^{i}=\left\{ \begin{aligned} 0 if exists j\in{{(Q}_{i}\cup i)}^{c}\mathrm{or}N_{i}{,G}_{k}^{j}=1,G_{k}^{i}\neq1 \\ S_{k}^{i}\mathrm{if}{exists j\in{{(Q}_{i}\cup i)}^{c}\mathrm{or}N_{i}, G}_{k}^{j}=G_{k}^{i}=1 \\ S_{k+1}^{i} \mathrm{others} \end{aligned}for i=1,2\cdots2^{n} \right.-1$. (S2)

In this case, cell cannot die but more cells get to partially reprogrammed state from lower levels (for example Fig S3 (a)). Thus, we couldn’t generally say that as reprogramming factors will activate a module randomly, the cell reaches one of the cell types from state ***ε*** with equal probability. In particular, the cell will not be reprogrammed but stay in state ***ɛ*** if reprogramming factors activate ESC module (an example shown in Fig S3 (b)), which is different from the original model and limits the reprogramming efficiency. Module states transition follows (2) and (S2); the new transition probability between cell states is shown in Fig S3(c). In the new Markov chain, all cells get to iPS state eventually but it takes a long time, about 8000 cell cycles, for nearly all the cells reprogrammed. Reprogramming efficiency accelerates a lot, about 1.1% in 20 cell cycles (Fig S2(b)) since the cell is free from death.

## Simulating MET in reprogramming

In reprogramming of MEF, the morphology change of cell is remarkable, during which fibroblast will change to tightly arranged round cells, inferring that mesenchymal to epithelial transition (MET) takes place [13]. EMT is an important step in gastrulation [21]. We assumed that there are four levels in the lineage tree (shown as Fig S4). Cells in the first level have specific mesenchymal markers, including several cell types in fibroblast. Cells dedifferentiate into level 2 expressing mesenchymal markers, including mesoderm. Then, MET happens when cells dedifferentiate into level 3 and cells in level 3 express epithelial markers. The fourth level is ESC in which some of epithelial markers related to pluripotency express, for example *E-cadherin* [13]. In the above section, we have already calculated the gene expression change in level 2 and level 3 (shown as the first picture in Fig 5 in the main text). Then we collected the expression data of some typical mesenchymal and epithelial markers in reprogramming (see Method). Besides, we normalized the expression data into [0,1] by $Y^{\mathrm{nor}}=\frac{Y-Y_{\min}}{Y_{\max}-Y_{\min}}$,$Y_{\min}$,$Y_{\max}$ are the minimum and maximum expression values of gene Y among different reprogramming days respectively. Some mesenchymal markers highly expressed in fibroblast such as *Snai2*, *vitronectin* decrease continually as genes in level 1. While others such as *FSP1*, *N-cadherin*, *fibronectin* change in the similar pattern as genes in level 2. The average peak time of these genes is 7 d (shown in Fig S5(a)), which is the same as the peak time of genes in level 2 (shown as the green curve in the first picture in Fig 5 in the main text). Some epithelial markers highly expressed in ESC increases continually such as *E-cadherin*, *claudin 10*. While others such as *Mucin-1*, *Occludins*, *keratin-8*, *claudin 3*, change in the similar pattern as genes in level 3. The average peak time of these genes is 12 d (shown in Fig S5(b)), which is the same as the peak time of genes in level 3 (shown as the red curve in the first picture in Fig 5 in the main text).

The reprogramming model can simulate MET in reprogramming process. These results may verify the existence of MET in reprogramming.

## The choice of parameters in reprogramming Ising model and the relationship of reprogramming Ising model and SRM model

Suppose repression is stronger than activation and epigenetic state is more variable than genetic state [2], then N<M<D<E. In the simulation, we assumed KT=1, E=50, D=20, M=8, N=5, F_1_=4, F_2_=16, if temperature rises fourfold, the restriction among nodes in the cell loses its force. Thus, the cell type conversion will just follow the reprogramming factors’ binding nodes, the efficiency of reprogramming will be much higher and the route of cell type conversion is pure random.

To show cell type transition explicitly and discuss the parameter range, we analyzed the dynamics supposing the cell lineage tree has 2 levels. Then there are only 3 nodes numbering 1 to 3 from top and leftmost. Initially, $G_{2}^{\mathrm{cell}}=1,G_{1}^{\mathrm{cell}}=G_{3}^{\mathrm{cell}}=0$. Suppose KT=1 and reprogramming factors active node j and repress node i, then:

$$<S_{1}>=\frac{\begin{aligned} sinh(-M\bullet{(G}_{2}^{\mathrm{cell}}{+G}_{3}^{\mathrm{cell}})+N\bullet G_{1}^{\mathrm{cell}}{-F}_{1}\bullet\delta(i,1)+F_{2}\bullet\delta(j,1)) \end{aligned}}{cosh(-M\bullet{(G}_{2}^{\mathrm{cell}}{+G}_{3}^{\mathrm{cell}})+N\bullet G_{1}^{\mathrm{cell}}{-F}_{1}\bullet\delta(i,1)+F_{2}\bullet\delta(j,1))+2}$$

$<S_{2}>=\frac{sinh(-M\bullet G_{3}^{\mathrm{cell}}+N\bullet G_{2}^{\mathrm{cell}}{-F}_{1}\bullet\delta\left( i,2 \right)+F_{2}\bullet\delta\left( j,2 \right))}{cosh(-M\bullet G_{3}^{\mathrm{cell}}+N\bullet G_{2}^{\mathrm{cell}}{-F}_{1}\bullet\delta\left( i,2 \right)+F_{2}\bullet\delta\left( j,2 \right))+2}$

$$<S_{3}>=\frac{sinh(-M\bullet G_{2}^{\mathrm{cell}}+N\bullet G_{3}^{\mathrm{cell}}{-F}_{1}\bullet\delta\left( i,3 \right)+F_{2}\bullet\delta\left( j,3 \right))}{cosh(-M\bullet G_{2}^{\mathrm{cell}}+N\bullet G_{3}^{\mathrm{cell}}{-F}_{1}\bullet\delta\left( i,3 \right)+F_{2}\bullet\delta\left( j,3 \right))+2}$$

As reprogramming factors only repress node with open chromatin state and only $S_{2}>0$ initially, i=2.

If$j=1,i=2$, $<S_{1}>=\frac{\begin{aligned} e^{-M+F_{2}}-e^{M-F_{2}} \end{aligned}}{e^{-M+F_{2}}+e^{M-F_{2}}+1}$ $<S_{2}>=\frac{\begin{aligned} e^{N-F_{1}}-e^{-N+F_{1}} \end{aligned}}{e^{N-F_{1}}+e^{-N+F_{1}}+1}$ $<S_{3}>=\frac{\begin{aligned} e^{-M}-e^{M} \end{aligned}}{\begin{aligned} e^{-M}+e^{M}+1 \end{aligned}}$. If ${N-F}_{1}>0 \mathrm{and} F_{2}\gg M\gg0, <S_{3}>\approx-1 <S_{1}>\approx1$ $0< <S_{2}>$<1

If$j=2,i=2$, $<S_{1}>=\frac{\begin{aligned} e^{-M}-e^{M} \end{aligned}}{\begin{aligned} e^{-M}+e^{M}+1 \end{aligned}}\approx-1$

$<S_{2}>=\frac{\begin{aligned} e^{N-F_{1}+F_{2}}-e^{-N+F_{1}-F_{2}} \end{aligned}}{e^{N-F_{1}+F_{2}}+e^{-N+F_{1}-F_{2}}+1}\approx1$ as $F_{2}\gg0 and {N-F}_{1}>0$

$<S_{3}>=\frac{\begin{aligned} e^{-M}-e^{M} \end{aligned}}{\begin{aligned} e^{-M}+e^{M}+1 \end{aligned}}\approx-1$

If$j=3,i=2$, then $<S_{1}>=\frac{\begin{aligned} e^{-M}-e^{M} \end{aligned}}{\begin{aligned} e^{-M}+e^{M}+1 \end{aligned}}\approx-1$ 0<$<S_{2}>=\frac{\begin{aligned} e^{N-F_{1}}-e^{-N+F_{1}} \end{aligned}}{e^{N-F_{1}}+e^{-N+F_{1}}+1}<1$ $<S_{3}>=\frac{\begin{aligned} e^{-M+F_{2}}-e^{M-F_{2}} \end{aligned}}{e^{-M+F_{2}}+e^{M-F_{2}}+1}\approx1$

Then, the genetic states change. The probability of getting to state $\left( G_{1},G_{2},G_{3} \right)=(1,0,0)$ is:

$$P\left( G_{1}=1,G_{2}=0,G_{3}=0 | S_{1}^{\mathrm{cell}}=S_{1}, S_{2}^{\mathrm{cell}}=S_{2}, S_{3}^{\mathrm{cell}}=S_{3} \right)=\frac{e^{DS_{1}}}{e^{DS_{1}}+e^{DS_{2}}+e^{DS_{3}}+e^{D\left( S_{1}+S_{2} \right)-E}+e^{D\left( S_{2}+S_{3} \right)-E}+e^{D\left( S_{1}+S_{3} \right)-E}+e^{D\left( {S_{1}+S}_{2}+S_{3} \right)-3E}+1}$$

Assuming $E-D\sim D\gg0$and only keeping the first order minim of $e^{-D}$, the conditional probability in each scenario is:

$P\left( G_{1}=1,G_{2}=0,G_{3}=0 | S_{1}^{\mathrm{cell}}=1, S_{2}^{\mathrm{cell}}=S_{2}, S_{3}^{\mathrm{cell}}=-1 \right)\approx\frac{1}{1+e^{D{(S}_{2}-1)}+e^{DS_{2}-E}+e^{-D}}$ (S3)

$P\left( G_{1}=1,G_{2}=0,G_{3}=0 | S_{1}^{\mathrm{cell}}=-1, S_{2}^{\mathrm{cell}}=1, S_{3}^{\mathrm{cell}}=-1 \right)\approx{O(e}^{-2D})\approx0$ (S4)

$P\left( G_{1}=1,G_{2}=0,G_{3}=0 | S_{1}^{\mathrm{cell}}=-1, S_{2}^{\mathrm{cell}}=S_{2}, S_{3}^{\mathrm{cell}}=1 \right)\approx\frac{1}{e^{D{(S}_{2}+1)}+e^{2D}}\approx{O(e}^{-2D})\approx0$ (S5)

$$P_{1}\left( G_{1}=1,G_{2}=0,G_{3}=0 \right)\approx\frac{1}{3}\cdot(1-e^{D{(S}_{2}-1)}-e^{DS_{2}-E}-e^{-D})$$

If $D{(1-S}_{2})=D\cdot\frac{\begin{aligned} 1+2\cdot e^{-N+F_{1}} \end{aligned}}{e^{N-F_{1}}+e^{-N+F_{1}}+1}>D\cdot e^{-N+F_{1}}\gg0$, $P_{1}\left( G_{1}=1,G_{2}=0,G_{3}=0 \right)\approx\frac{1}{3}(1+o(1))$.Symmetrically, $P_{1}\left( G_{1}=0,G_{2}=1,G_{3}=0 \right)\approx P_{1}\left( G_{1}=0,G_{2}=1,G_{3}=0 \right)\approx\frac{1}{3}$.Thus, the probability of getting to other configuration is $o(1)$.

In the next cell cycle, we summed the probability of dedifferentiation on condition of different configuration of $\left( G_{1}^{\mathrm{cell}},G_{2}^{\mathrm{cell}},G_{3}^{\mathrm{cell}} \right)$:

$P\left( G_{1}=1,G_{2}=0,G_{3}=0 \right)={\sum_{\left( G_{1}^{\mathrm{cell}},G_{2}^{\mathrm{cell}},G_{3}^{\mathrm{cell}} \right)\neq\left( 1,0,0 \right)} P\left( G_{1}=1,G_{2}=0,G_{3}=0 | {S_{i}^{\mathrm{cell}}=<S}_{i}> \right)P}_{1}\left( G_{1}^{\mathrm{cell}},G_{2}^{\mathrm{cell}},G_{3}^{\mathrm{cell}} \right)+P_{1}\left( G_{1}=1,G_{2}=0,G_{3}=0 \right)$ (S6)

where${<S}_{1}>=\frac{\begin{aligned} sinh(-M\bullet{(G}_{2}^{\mathrm{cell}}{+G}_{3}^{\mathrm{cell}})+N\bullet G_{1}^{\mathrm{cell}}) \end{aligned}}{cosh(-M\bullet{(G}_{2}^{\mathrm{cell}}{+G}_{3}^{\mathrm{cell}})+N\bullet G_{1}^{\mathrm{cell}})+2} <S_{2}>=\frac{sinh(-M\bullet G_{3}^{\mathrm{cell}}+N\bullet G_{2}^{\mathrm{cell}})}{cosh(-M\bullet G_{3}^{\mathrm{cell}}+N\bullet G_{2}^{\mathrm{cell}})+2}$ $<S_{3}>=\frac{sinh(-M\bullet G_{2}^{\mathrm{cell}}+N\bullet G_{3}^{\mathrm{cell}})}{cosh(-M\bullet G_{2}^{\mathrm{cell}}+N\bullet G_{3}^{\mathrm{cell}})+2}$.

If $\left( G_{1}^{\mathrm{cell}},G_{2}^{\mathrm{cell}},G_{3}^{\mathrm{cell}} \right)=(1,0,0)$ , $<S_{1}>=\frac{e^{N}-e^{-N}}{e^{N}+e^{-N}+1}\approx1$when $N\gg0 ,<S_{2}>=0<S_{3}>=0$.Thus, the cell dedifferentiates one level. If$\left( G_{1}^{\mathrm{cell}},G_{2}^{\mathrm{cell}},G_{3}^{\mathrm{cell}} \right)=\begin{matrix} (0,1,0) & (1,1,0) \\ (0,0,1) & (1,0,1) \end{matrix}$, then $\left\{ \begin{aligned} <S_{1}>=\frac{e^{N-M}-e^{-N+M}}{e^{N-M}+e^{-N+M}+1}\mathrm{or}\frac{e^{-M}-e^{M}}{e^{-M}+e^{M}+1}=-1 \\ <S_{2}>=\pm1 \\ <S_{3}>=\mp1 \end{aligned} \right.$ when $M\gg N$. As (S4), the probability of cell dedifferentiating is ${O(e}^{-2D})$ in these cases, so we neglected this term. If$\left( G_{1}^{\mathrm{cell}},G_{2}^{\mathrm{cell}},G_{3}^{\mathrm{cell}} \right)=\left( 1,1,1 \right) or (0,1,1)$, then $<S_{1}>\approx<S_{2}>\approx<S_{3}>\approx-1$ $P\left( G_{1}=1,G_{2}=0,G_{3}=0 | S_{1}^{\mathrm{cell}}=-1, S_{2}^{\mathrm{cell}}=-1, S_{3}^{\mathrm{cell}}=-1 \right)={O(e}^{-D})$. On the other hand, $P_{1}\left( 1,1,1 \right)\mathrm{and}P_{1}(0,1,1)$ is $o(1)$, as in (S6), the probability of cell dedifferentiation in this case is ${o(e}^{-D})$ and we neglected it. If $\left( G_{1}^{\mathrm{cell}},G_{2}^{\mathrm{cell}},G_{3}^{\mathrm{cell}} \right)=(0,0,0)$, then $<S_{1}>\approx<S_{2}>\approx<S_{3}>\approx0$ and $P\left( G_{1}=1,G_{2}=0,G_{3}=0 | S_{1}^{\mathrm{cell}}=0, S_{2}^{\mathrm{cell}}=0, S_{3}^{\mathrm{cell}}=0 \right)\approx\frac{1}{4}$. Since $P_{1}\left( 0,0,0 \right)\approx e^{-D}$, the probability of cell dedifferentiation in this case is ${1/4\cdot e}^{-D}$ as in (S6).

Therefore, the probability of a cell reprogrammed in two cell cycles is $\frac{1}{3}\cdot(1-e^{D{(S}_{2}-1)}-e^{DS_{2}-E}-e^{-D})$+${\frac{1}{4}\cdot e}^{-D}=\frac{1}{3}(1+o(1))$, which is consistent with the 2-level SRM model. Then, the proportion of cells reprogrammed in 2k cell cycles is $1-\left( \frac{2}{3} \right)^{k}$.

In sum, suppose KT=1, then when the parameters are in the range ${N-F}_{1}>0 ,F_{2}\gg M\gg N\gg0 , E-D\sim D\gg0$ $D\cdot e^{-N+F_{1}}\gg0$, the reprogramming is rare and the energy model is consistent with SRM model. That is, the reprogramming Ising model returns to SRM model when the “temperature” is low.

## The expression of some cell type specific genes in different tissues

Each cell type can be represented by several cell type specific genes, which highly express in such cell type but express lowly in others. For example, *Oct4* highly express in ESCs and its expression decrease as differentiation (Fig S6, S7). *Dppa1*, a pluripotency-related gene and downstream gene of *Oct4* can also represent ESC which only highly express in blastocytes (Fig S8(a)). *Snai2*, a mesenchymal marker, shows tissue specific expression and can be contained in the MEF module (Fig S8(b)). *Gata6*, an endodermal transcription factor, also can be a cell type marker (Fig S8(c)).


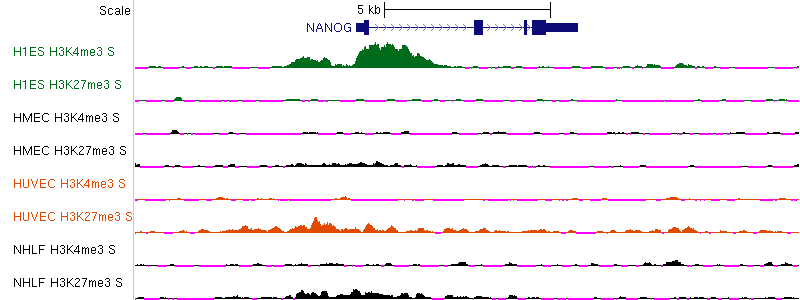


(a)


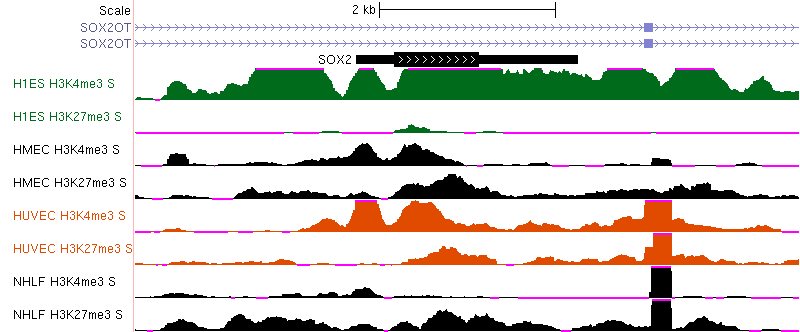


(b)


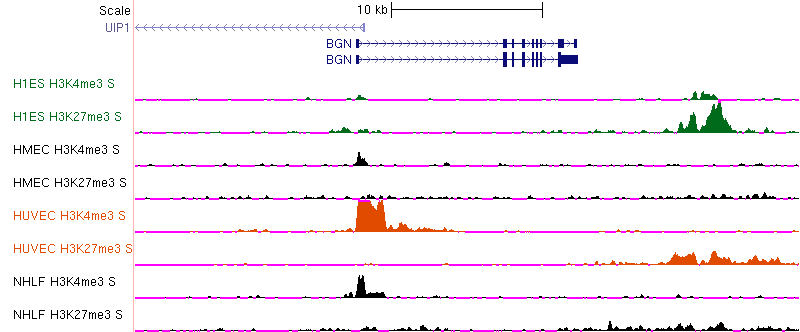


(c)

## Figure S1 - H3K4me3 and H3K27me3 at *Sox2*, *NANOG*, *BGN* locus in different human cell type

1. *Nanog* shows high level of H3K4 in ESCs, but some degree of H3K27me3 (significant peaks, p<10^-10^) show in other cell types.
2. *Sox2* shows high level of H3K4 and low level of H3K27 tri-methylation in ESCs, but shows H3K4me3 and H3K27me3 in other cell types from three different germ layers.
3. *BGN* (*biglycan*) is related with skeletal system development and extracellular matrix. Its promoter has some H3K4me3 and H3K27me3 in ESCs; enriched for H3K4me3 in HUVEC.

HMEC: human mammary epithelial cells from ectoderm. H1ES: embryonic stem cells from inner cell mass. NHLF: normal human lung fibroblasts from endoderm. HUVEC: umbilical vein endothelial cells from mesoderm. The picture is from ENCODE Histone Modifications [22] by Broad Institute ChIP-seq track in <http://genome.ucsc.edu>. The ChIP-seq data [14,16] were generated at the [Broad Institute](http://www.broadinstitute.org/science/projects/epigenomics/epigenomics-initiative#_blank) and in the [Bradley E. Bernstein lab](http://csb.mgh.harvard.edu/bradley_bernstein#_blank) at the Massachusetts General Hospital/Harvard Medical School. Data generation and analysis was supported by funds from the NHGRI, the Burroughs Wellcome Fund, Massachusetts General Hospital and the Broad Institute.

(a)

(b)

## Figure S2 - Reprogramming rate increases by specific transcription factors knockdown or DNA methylation inhibition

1. Reprogramming rate increases a little to 0.027% by specific transcription factors knockdown.
2. Reprogramming rate is much higher upon DNA methylation inhibition.

(a)

(b)

(c)

## Figure S3 - Partially reprogrammed state and transition probability when inhibition of DNA methylation

1. Initial partially reprogrammed state: the picture shows the state of all the modules arranged in the module lineage tree.
2. After reprogramming factors activate ESC module, the cell stays in partially reprogrammed state since gene expression from ESC module cannot make the epigenetic state of the module in the bottom close.
3. The new Markov chain under the condition of global DNA demethylation. The transition probabilities are the number indicated in the picture divided by 15.

## Figure S4 - Simulating MET using Markov Model

(a)

(b)

## Figure S5 - Some mesenchymal and epithelial Markers change similarly as Level 2 and Level 3 respectively

1. mesenchymal markers expression variation curve (b) epithelial markers expression variation curve


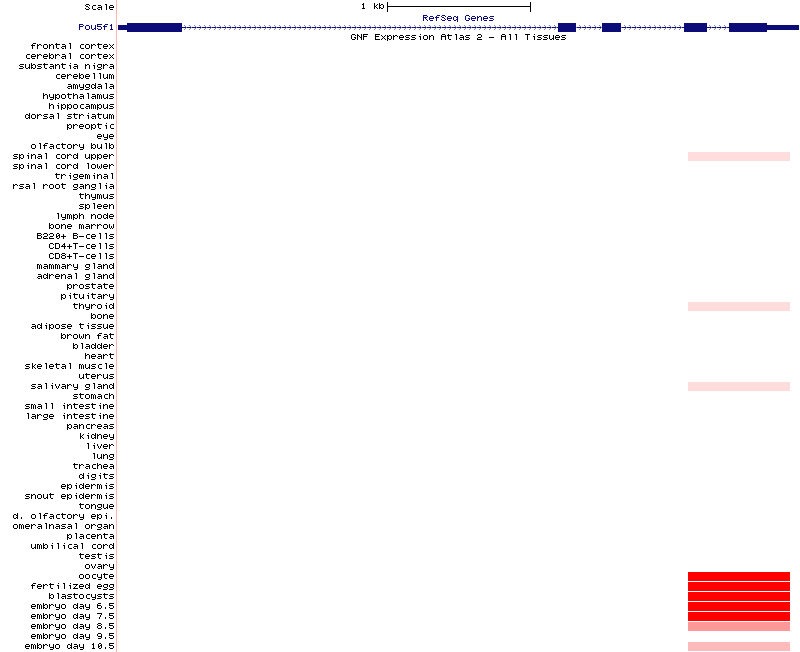


## Figure S6 - *Oct4* only express highly in fertilized egg, blastocysts and early embryos

The picture is from GNF Atlas 2 track [23] in <http://genome.ucsc.edu>. Red is over expression while blue is under expression.

## Figure S7 - The expression of *Pou5f1*(*Oct4*) decreases as differentiation

The original data is from GSE3231 [24] and is normalized by Dchip [18].


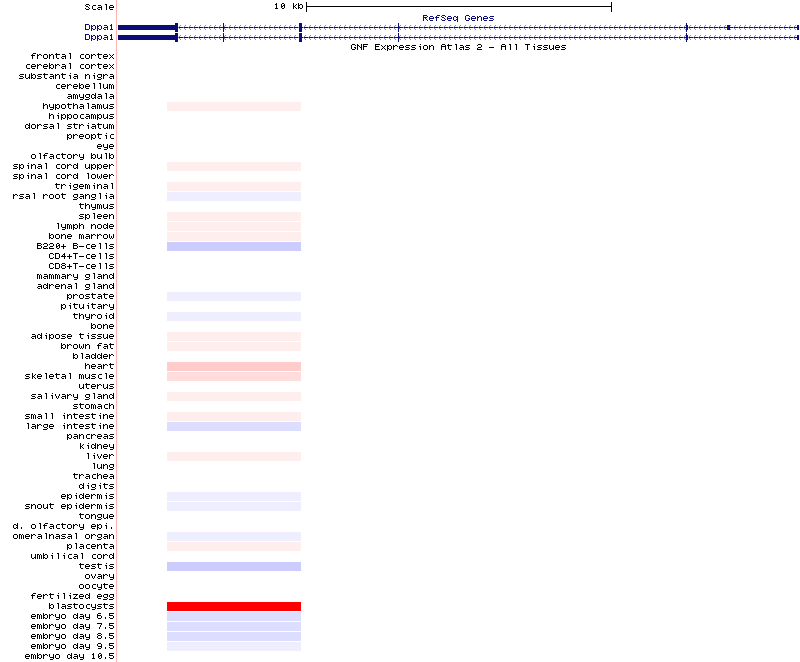


(a)


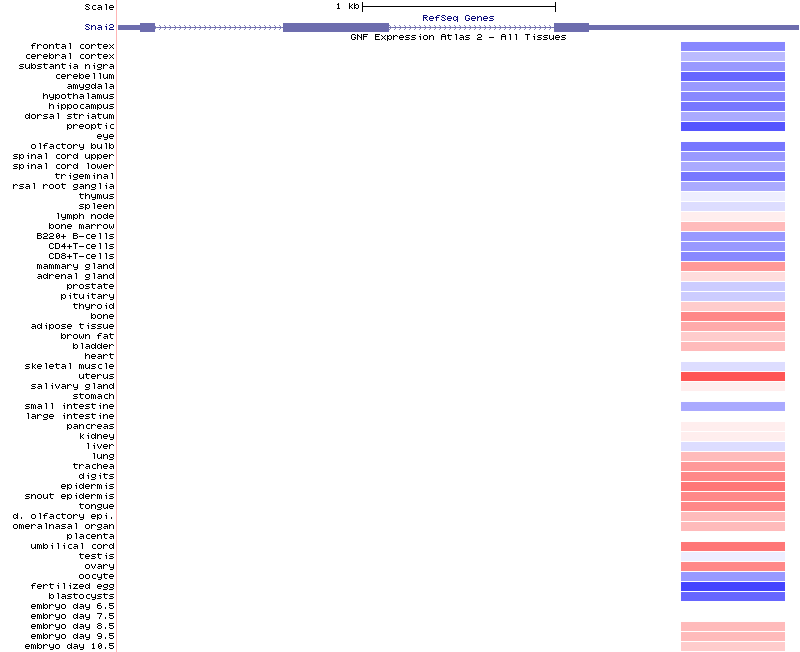


(b)


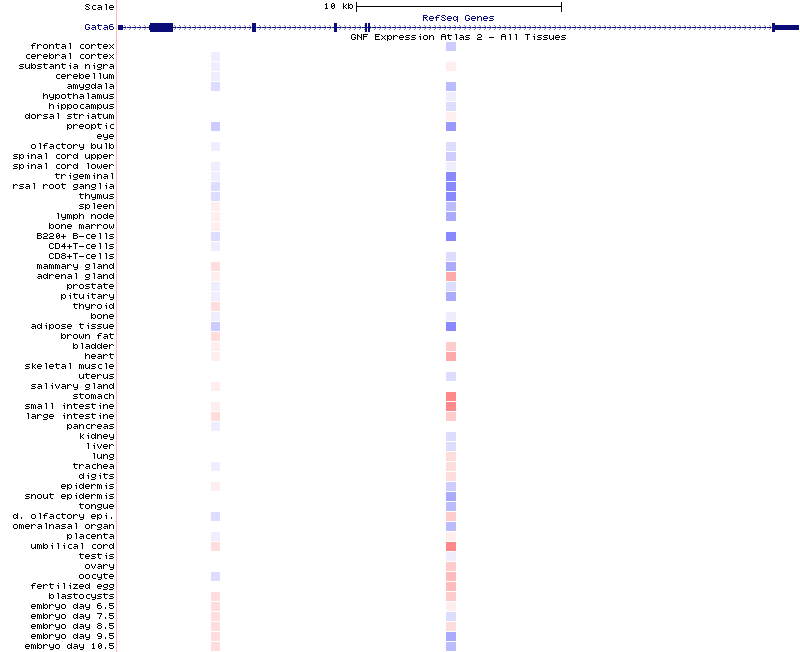


(c)

## Figure S8 - *Dppa1*, *Snai2* and *Gata6* show tissue specific expression

1. *Dppa1* only highly express in blastocysts.
2. *Snai2* shows tissue specific expression.
3. *Gata6* shows tissue specific expression.

The picture is from GNF Atlas 2 track [23] in <http://genome.ucsc.edu>. Red is over expression while blue is under expression.

111

| 8→4→2→3→1 | 8→4→2→3→1 | 8→4→2→1 |
| --- | --- | --- |
| 8→4→2→1 | 8→8→4→2→1 | 8→4→4→2→1 |
| 8→4→2→1 | 8→4→5→5→2→1 | 8→4→2→2→1 |

## Figure S9 - Successfully reprogrammed Cells share similar Trajectories

The cell type is numbered from 1 to 15 from top and left of the cell lineage tree. The black arrows are one of the trajectories of successfully reprogrammed cell. The list below shows the trajectories of the 9 successfully reprogrammed cells.


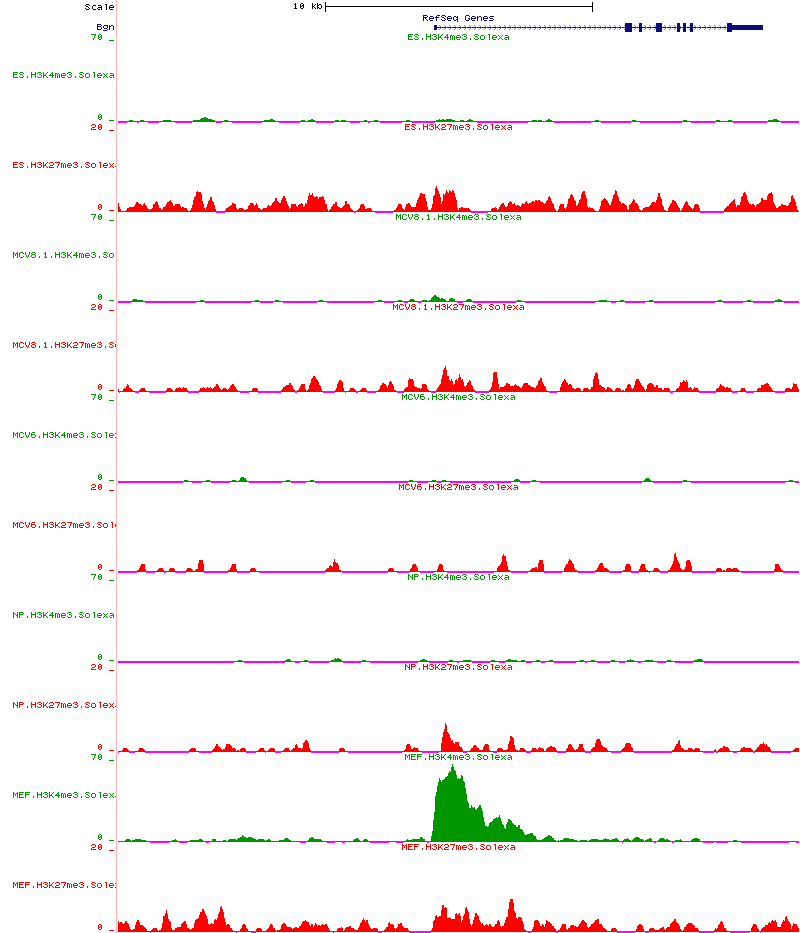


## Figure S10 - The epigenetic state change at *BGN* locus in Reprogramming

*BGN* shows high H3K4me3 but shows some H3K4me3 and H3K27me3 in iPS, referring to somatic cell curve in Fig 6 in the main text. ES: embryonic stem cells. MEF: mouse embryonic fibroblasts. NP: neuronal precursor cells. MCV6: partially reprogrammed cells. MCV8.1: a clone of iPSCs.

The data is from [3] and the picture is from in <http://genome.ucsc.edu>.


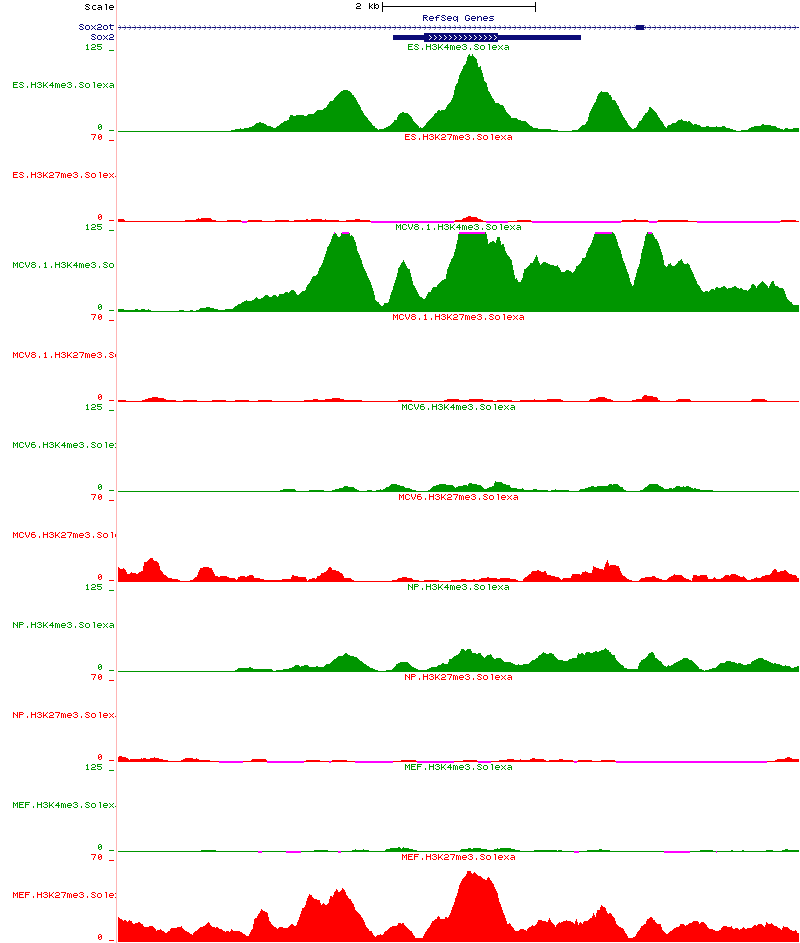


(a)


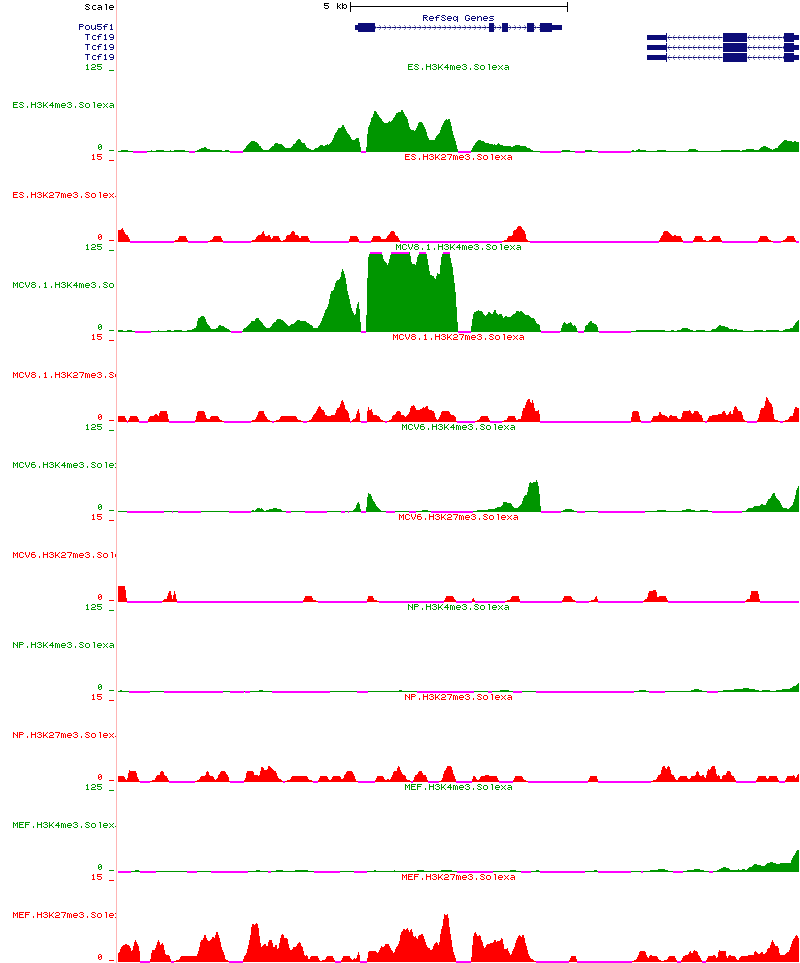


(b)


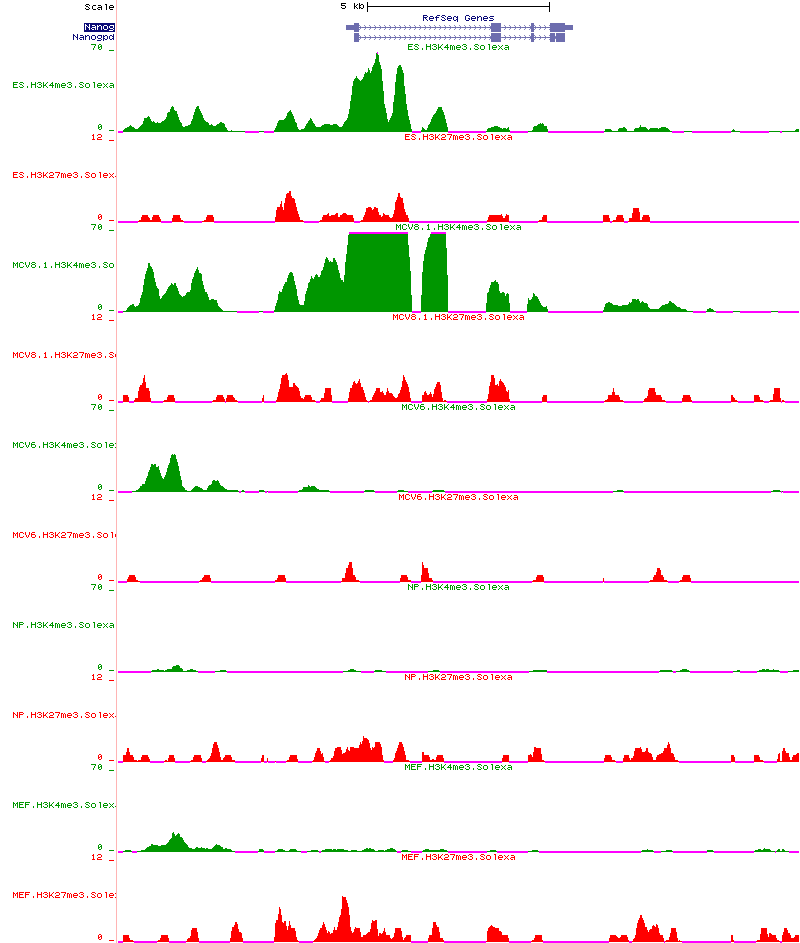


(c)

## Figure S11 - The epigenetic state change at *Sox2*, *Oct4* and *Nanog* loci in Reprogramming

*Sox2*, *Oct4* and *Nanog* shows high level of H3K4me3 (open state) in ESC and iPSC, high level of H3K27me3 (close state) in MEF and mediate levels of H3K27me3 and H3K4me3 in MCV6 and NP, referring to ESC curve in Fig 6 in the main text. The abbreviation is the same as Fig S10.

The data is from [3] and the picture is from in <http://genome.ucsc.edu>.

## Table S1 - Reprogramming rates of different Number of levels in the cell lineage tree

| Number of levels | First level | Second level | Third level | Fourth level | Fifth level | Sixth level |
| --- | --- | --- | --- | --- | --- | --- |
| 4 | 0.024% | 0.26% | 3.13% | 100% | — | — |
| 5 | 0.0005% | 0.014% | 0.37% | 0.67% | 100% | — |
| 6 | $\approx$0 | 0.0015% | 0.086% | 0.098% | 0.16% | 100% |
| 7 | $\approx$0 | 0.0002% | 0.021% | 0.024% | 0.025% | 0.040% |

Although the number of levels is different, the reprogramming rate of the fourth level from ESC is similar.

## References

21. Jean Paul Thiery, Jonathan P. Sleeman: **Complex networks orchestrate epithelial–mesenchymal transitions**. *Nature Rev Mol Cell Biol* 2006, **7**: 131-142.

22. ENCODE Project Consortium: [**Identification and analysis of functional elements in 1% of the human genome by the ENCODE pilot project.**](http://www.ncbi.nlm.nih.gov/pubmed/17571346) *Nature* 2007, **447(7146)**:799-816.

23. [Andrew I. Su](http://www.pnas.org/search?author1=Andrew+I.+Su&sortspec=date&submit=Submit), [Tim Wiltshire](http://www.pnas.org/search?author1=Tim+Wiltshire&sortspec=date&submit=Submit), [Serge Batalov](http://www.pnas.org/search?author1=Serge+Batalov&sortspec=date&submit=Submit), [Hilmar Lapp](http://www.pnas.org/search?author1=Hilmar+Lapp&sortspec=date&submit=Submit), [Keith A. Ching](http://www.pnas.org/search?author1=Keith+A.+Ching&sortspec=date&submit=Submit), [David Block](http://www.pnas.org/search?author1=David+Block&sortspec=date&submit=Submit), [Jie Zhang](http://www.pnas.org/search?author1=Jie+Zhang&sortspec=date&submit=Submit), [Richard Soden](http://www.pnas.org/search?author1=Richard+Soden&sortspec=date&submit=Submit), [Mimi Hayakawa](http://www.pnas.org/search?author1=Mimi+Hayakawa&sortspec=date&submit=Submit), [Gabriel Kreiman](http://www.pnas.org/search?author1=Gabriel+Kreiman&sortspec=date&submit=Submit), [Michael P. Cooke](http://www.pnas.org/search?author1=Michael+P.+Cooke&sortspec=date&submit=Submit), [John R. Walker](http://www.pnas.org/search?author1=John+R.+Walker&sortspec=date&submit=Submit), [John B. Hogenesch](http://www.pnas.org/search?author1=John+B.+Hogenesch&sortspec=date&submit=Submit): [**A gene atlas of the mouse and human protein-encoding transcriptomes**](http://www.pnas.org/cgi/content/abstract/101/16/6062)**.** *Proc Natl Acad Sci* *U S A* 2004, **101(16)**:6062-7.

24. Kagnew Hailesellasse Sene, Christopher J Porter, Gareth Palidwor, Carolina Perez-Iratxeta, Enrique M Muro, Pearl A Campbell, Michael A Rudnicki, Miguel A Andrade-Navarro: **Gene function in early mouse embryonic stem cell differentiation**. *BMC Genomics* 2007, **8**: 85.
